# Austrian Syndrome: The Forgotten Triad of a Complex Condition in an Antibiotic Era

**DOI:** 10.7759/cureus.32106

**Published:** 2022-12-01

**Authors:** Neelanjana Pandey, Yakoot Khan, Tobechukwu Okobi, David Uhomoibhi, Adesewa Abolurin, Ololade A Akinlabi, Ebikiye Angaye, Miguel A Rodriguez Guerra, Timothy Vittorio

**Affiliations:** 1 Internal Medicine, BronxCare Health System, Bronx, USA; 2 Internal Medicine, BronxCare Health System, New York, USA; 3 Internal Medicine, Georgetown University, Bronx, USA; 4 General Practice, University of Lagos College of Medicine, Lagos, NGA; 5 Family Medicine, Bowen University, Ogbomosho, NGA; 6 Family Medicine, Diete-Koki Memorial Hospital, Yenagoa, NGA; 7 Cardiology, Montefiore Medical Center, Albert Einstein College of Medicine, Bronx, USA; 8 Cardiology, BronxCare Health System, Bronx, USA

**Keywords:** pneumonia, meningitis, endocarditis, streptococcus pneumonia, osler's triad, austrian syndrome

## Abstract

Osler’s triad, an alternative term for Austrian syndrome, has a complex pathology comprising of pneumonia, meningitis, and endocarditis, all of which are caused by the hematogenous dissemination of *Streptococcus pneumoniae*. It can affect multiple organ systems, resulting in this rare but complex triad. With the advent of antibiotics, the incidence and severity of the disease have reduced remarkably. However, it still remains a lethal disease requiring early diagnosis and prompt treatment.

We present the case of a 58-year-old male, with a past medical history of cerebrovascular accident and alcohol dependency, who presented with altered mental status, flu-like symptoms, fever, and vomiting. The patient was initially diagnosed with meningoencephalitis and pneumonia from *Streptococcus pneumoniae*, and despite adequate antibiotic treatment, he subsequently developed bacterial endocarditis, requiring valve replacement.

Austrian syndrome is an uncommon life-threatening condition with a high mortality rate. Its outcome depends on an early diagnosis to establish antimicrobial therapy and to define potential surgical approach in order to improve the outcome of the patient.

## Introduction

Austrian syndrome is described as a triad of pneumonia, endocarditis, and meningitis, with a strong association with heavy alcohol abuse. The leading cause of Austrian syndrome is *Streptococcus pneumoniae*. Other risk factors are old age, immunosuppression, and asplenism. This complex triad was first described by Sir William Osler in 1881 but was first published by Robert Austrian in 1957. Patients diagnosed with Austrian syndrome usually present with symptoms of various organ systems involved including, but not limited to, fever, altered mentation, shortness of breath, productive cough, and chest pain.

It is paramount to diagnose Austrian syndrome as early as possible to increase the chances of survival and reduce the likelihood of complications. Bacterial culture is the main method of diagnosis with ancillary investigations including X-ray, and an echocardiogram [1].

We describe an interesting and rare case of Austrian syndrome consisting of a triad of endocarditis, meningitis, and pneumonia caused by *Streptococcus pneumoniae*.

## Case presentation

The patient is a 58-year-old male who was brought to our emergency room (ER) with altered mental status, fever, chills, and vomiting after three weeks of flu-like symptoms that improved on symptomatic treatment. Past medical history was significant for a cerebrovascular accident and alcohol dependence.

In the ER, Glasgow coma scale (GCS) was 7 (E2V1M5) with nuchal rigidity, he was febrile (103.6 F), tachycardic (127 bpm), and tachypneic (24 rpm), with elevated blood pressure (162/56 mmHg). Laboratory results showed leukocytosis with predominant neutrophilia, hyponatremia, lactic acidosis, elevated pro-BNP (pro-brain natriuretic peptide) (Table 1).

**Table 1:** Relevant laboratory results on initial presentation Abbreviations: pCO_2_, partial pressure of carbon dioxide; pO_2_, partial pressure of oxygen

Parameters	Results	Reference
Hemoglobin	12.1	12.0-16.0 (g/dL)
Platelet	155	150-400 (k/uL)
White blood cell	11.6	4.8-10.8 (k/uL)
Neutrophil count	10.5	1.5-8.0 (k/uL)
Neutrophil %	90.3	40.0- 70.0 (%)
Chemistry		
Sodium	125	135-145 (mEq/L)
Potassium	3.7	3.5-5.0 (mEq/L)
Blood urea nitrogen (serum)	20	8-26 (mg/dL)
Creatinine	1.0	0.5-1.5 (mg/dL)
Pro-brain natriuretic peptide	1433	0-125 (pg/mL)
Lactic acid	2.3	0.5-1.6 (mmol/L)
Blood gas		
pH	7.54	7.350-7.450
pCO_2_	28.4	35.0-45.0 (mmHg)
pO_2_	34.2	83.0-108.0 (mmHg)
Serum bicarbonate	22	24-30 (mEq/L)

**Figure 1:**
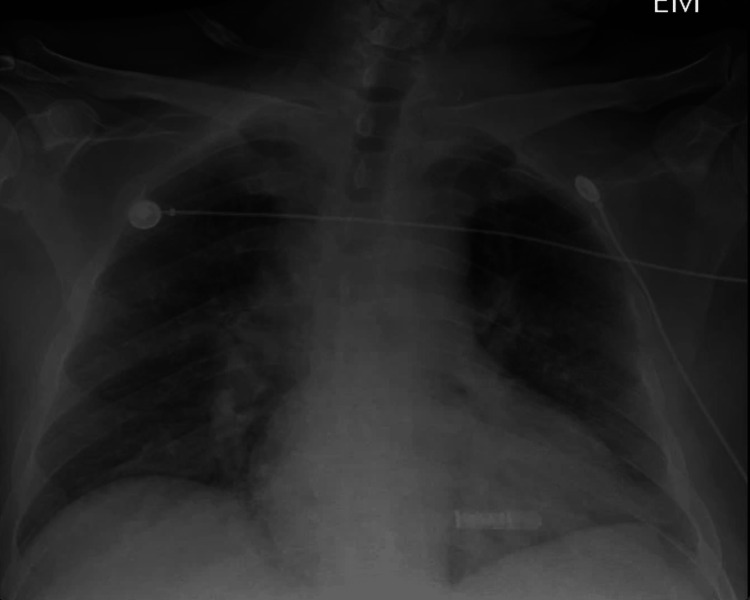
Chest X-ray showing diffuse interstitial pulmonary edema

**Figure 2:**
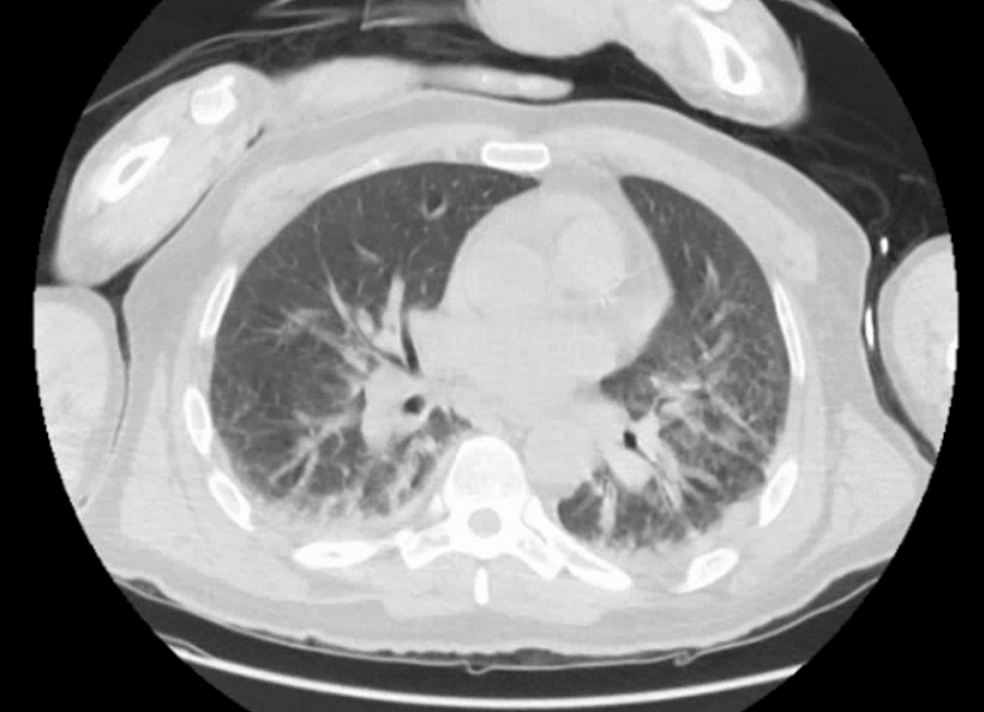
Chest CT showing bilateral bibasilar consolidations consistent with pneumonia and bilateral trace pleural effusion

The chest X-ray showed features consistent with diffuse interstitial pulmonary edema (Figure 1). The chest CT showed bilateral bibasilar consolidations consistent with pneumonia, bilateral trace pleural effusions, cardiomegaly, and splenomegaly (Figure 2).

The patient was started on ceftriaxone, ampicillin, acyclovir, vancomycin, dexamethasone, and oxygen therapy, and was admitted to the intensive care unit (ICU). CT of the head was negative, but chronic lacunar infarcts on the left thalamus were noticed. Lumbar puncture was compatible with bacterial meningitis (Table 2).

**Table 2:** CSF results on initial admission Abbreviations: CSF, cerebrospinal fluid; HSV PCR, herpes simplex virus polymerase chain reaction; RBC, red blood cell; WBC, white blood cell

CSF parameters	Result	Reference/unit
Color	Light yellow	Colorless
Appearance	Cloudy	Clear
RBC count	170	<10 RBCs/uL
WBC count	158	<5 PMNs/uL
Segmented cell count (%)	88	%
Monocyte count (%)	3	%
Lymphocyte count (%)	9	%
Glucose (mg/dL)	45	40-70 (mg/dL)
Lactic acid (mmol/L)	14.0	0.6-2.2 (mmol/L)
Protein (mg/dL)	267	14-45 (mg/dL)
Albumin (g/dL)	2.6	3.5-5.2 (g/dl)
CSF bacterial antigen	*Haemophilus influenzae B*: negative	
	*Streptococcus pneumoniae*: negative	
	Group B Streptococcus: negative	
	*Neisseria meningitidis* C/W135: negative	
	*Neisseria meningitidis* A/Y: negative	
	*Neisseria meningitidis* B/E coli K1: negative	
	Cryptococcal antigen: not detected	
CSF culture	No organisms or white blood cells seen	
CSF gram stain	No organisms seen. Many polymorphonuclear leukocytes seen	
CMV	<2.3	<2.3 (log IU/mL)
CMV antibody (<200)	<200 IU/mL	<200 (IU/mL)
Fungal culture	No fungi isolated in 28 days	
HSV PCR	Not detected	

The urine streptococcal pneumonia antigen was positive and blood culture grew *Streptococcus pneumonia* (Table 3). Antibiotics was tailored to the organism isolated on blood culture (IV ceftriaxone).

**Table 3:** Microbiology Abbreviation: AFB, acid-fast bacilli

Parameter	Results
Cryptococcal antigen	Not detected
Blood culture	Growth of *Streptococcus pneumoniae*
Respiratory culture	Gram-positive cocci in pairs
Sputum culture	Negative
*Pneumocystis Jirovecii* (*Pneumocystis carinii*)	Not detected
AFB	No acid-fast bacilli seen
Viral culture	No virus isolated

The patient was also noted to have new-onset atrial fibrillation and was started on apixaban. Echocardiogram showed an ejection fraction (EF) of 66%, grade II diastolic dysfunction, severely elevated pulmonary artery pressure, and thickening of both mitral and aortic valves, with mild tricuspid and mitral regurgitation. During this course of admission, the patient showed signs of clinical improvement, completed his antibiotics course, and was subsequently discharged.

However, the patient returned to the ER a week later with complaints of severe shortness of breath of 1-day duration. In the ER, he was tachycardic (109 bpm), in severe respiratory distress with hypoxia, tachypneic with use of accessory muscles of respiration, and hypertensive (144/77 mmHg).

On examination, he had a holosystolic murmur at the third intercostal space, and on lung auscultation, there were bibasilar crackles and bilateral pitting edema. Labs showed leukocytosis, hyponatremia, and elevated pro-BNP (Table 4).

**Table 4:** Relevant laboratory results on re-admission Abbreviations: pCO_2_, partial pressure of carbon dioxide; pO_2_, partial pressure of oxygen

Parameters	Results	Reference (unit)
Hemoglobin	12.1	12.0-16.0 (g/dL)
Platelet	394	150-400 (k/uL)
White blood cell	17.9	4.8-10.8 (k/uL)
Neutrophil count	15.3	1.5-8.0 (k/uL)
Neutrophil %	85.8	40.0- 70.0 (%)
Chemistry		
Sodium	129	135-145 (mEq/L)
Potassium	5.0	3.5-5.0 (mEq/L)
Blood urea nitrogen (serum)	16	8-26 (mg/dL)
Creatinine	1.0	0.5-1.5 (mg/dl)
Pro-brain natriuretic peptide	6637	0-125 (pg/mL)
Lactic acid	5.0	0.5-1.6 (mmol/L)
Blood gas		
pH	7.2	7.350-7.450
pCO_2_	59.7	35.0-45.0 (mmHg)
pO_2_	22.9	83.0-108.0 (mmHg)
Serum bicarbonate	20	24-30 (mEq/L)

The patient was admitted to the ICU due to acute hypoxic respiratory failure with ARDS, and septic shock, and was placed on mechanical ventilator and started on broad-spectrum antibiotics.

Chest X-ray showed bilateral pleural effusions with bilateral perihilar airspace disease. Respiratory cultures showed gram-positive cocci in pairs.

Echocardiogram showed reduced EF (49%), right ventricular systolic pressure of 37%, and the presence of vegetations on the coronary and non-coronary cusps of the aortic valve consistent with endocarditis. The patient subsequently underwent emergent aortic valve replacement with a bioprosthetic valve and completed his antibiotics course. He continued to show features of clinical improvement. He was followed up at the clinic where his exercise tolerance improved as evidenced by an adequate exercise treadmill test.

## Discussion

Austrian syndrome, also known as Osler’s triad, is a form of invasive pneumococcal disease associated with fatal outcomes if not identified and treated early. It consists of a triad of pneumonia, meningitis, and endocarditis caused by *Streptococcus pneumonia* evidenced on cultures. It was first described by Sir William Osler in 1881 but was first published by Robert Austrian in 1957 [2].

The widespread use of antibiotics and pneumococcal vaccination has significantly decreased the incidence and severity of the disease from 10-15% to 3% in the pre-antibiotic era [1]. However, mortality from the disease could be 60% if it is not diagnosed early [3]. Risk factors include chronic alcoholism, male sex, advanced age, diabetes mellitus, chronic kidney disease, liver and pulmonary disease, asplenism, and other forms of immunosuppression [4]. Intravenous drug use has also been associated with Austrian syndrome [5].

Echocardiography is also crucial for the demonstration of endocarditis. Transthoracic echocardiogram (TTE) and transesophageal echocardiogram (TEE) are both important tools in visualizing the valves and identifying vegetative legions that may be present [6]. As early surgical intervention has been associated with decrease in mortality associated with the streptococcal endocarditis, usually TTE is performed first, and once this condition is identified, then TEE is required [7].

Management of Austrian syndrome involves promptly identifying and diagnosing the triad of pneumonia, endocarditis, and meningitis. The therapy requires multidisciplinary care by infectious disease, cardiology, cardiac surgery, neurology, and pulmonary specialists to start an early antimicrobial therapy, monitoring of response to therapy, and optimizing the patient for a possible surgical intervention. Most of these patients with streptococcal endocarditis have aortic valve involvement (75%), with most of them requiring valve replacements [8]. Two antibiotics with different anti-pneumococcal mechanisms of action is preferred for patients with known or suspected invasive pneumococcal infection and tailored to susceptibility results when available during which monotherapy can be used. Penicillin and third-generation cephalosporins with vancomycin have been used [9]. However, the choice of empiric antibiotics is dependent on local patterns of resistance and sensitivity results. With mortality rates as high as 60% if not diagnosed early [3], it is imperative to identify early enough this complex triad, initiate proper antibiotic treatment, and promptly manage complications.

Less than 1% of patients with endocarditis from *Streptococcus pneumonia* have the classic triad [10]. Our patient presented with typical risk factors (male and chronic alcoholic) and the disease triad. Despite adequate antibiotic therapy on his initial presentation, he developed endocarditis with aortic valve involvement requiring aortic valve replacement, prolonged antibiotic therapy, and had a positive outcome.

## Conclusions

Austrian syndrome is an uncommon life-threatening disease consisting of pneumonia, meningitis, and endocarditis, all of which are caused by the hematogenous dissemination of *Streptococcus pneumoniae*. It requires a high index of suspicion to establish a prompt broad-spectrum antibiotic therapy, followed by a guided work-up for a proper diagnosis, in order to reduce morbidity and the high mortality associated with this fatal condition.
