# Correction: Austrian Syndrome: The Forgotten Triad of a Complex Condition in an Antibiotic Era

**DOI:** 10.7759/cureus.c99

**Published:** 2023-02-03

**Authors:** Neelanjana Pandey, Yakoot Khan, Tobechukwu Okobi, David Uhomoibhi, Adesewa Abolurin, Ololade A Akinlabi, Ebikiye Angaye, Miguel A Rodriguez Guerra, Timothy Vittorio

**Affiliations:** 1 Internal Medicine, BronxCare Health System, Bronx, USA; 2 Internal Medicine, BronxCare Health System, New York, USA; 3 Internal Medicine, Georgetown University, Bronx, USA; 4 General Practice, University of Lagos College of Medicine, Lagos, NGA; 5 Family Medicine, Bowen University, Ogbomosho, NGA; 6 Family Medicine, Diete-Koki Memorial Hospital, Yenagoa, NGA; 7 Cardiology, Montefiore Medical Center, Albert Einstein College of Medicine, Bronx, USA; 8 Cardiology, BronxCare Health System, Bronx, USA

This article has been corrected at the request of the authors to change the following author’s name:

From: Miguel Rodriguez Guerra 

To: Miguel A. Rodriguez Guerra 

The authors deeply regret that this error was not identified and addressed prior to publication.

 

